# Extranodal marginal zone lymphoma of mucosa-associated lymphoid tissue of the ileum in an adult presenting with intussusception: a case report and literature review

**DOI:** 10.3389/fonc.2024.1395144

**Published:** 2024-06-11

**Authors:** Binlin Da, Juanjuan Zhang, Feng Zhu, Zhiming Wang, Yanqing Diao

**Affiliations:** Research Institute of General Surgery, Jinling Hospital, Affiliated Hospital of Medical School, Nanjing University, Nanjing, Jiangsu, China

**Keywords:** extranodal marginal zone B-cell lymphoma of the mucosa-associated lymphoid tissue, MALT lymphoma, Ileocolic intussusception, abdominal pain, case report

## Abstract

Extranodal marginal zone lymphoma of mucosa-associated lymphoid tissue (EMZL), also known as MALT lymphoma, is an extranodal multiorgan-invasive proliferative lymphoma composed of small B cells with variable morphology. It most commonly occurs in the digestive tract, with a high prevalence in the stomach, but EMZL originating in the small intestine is rare and lacks specificity in clinical manifestations, which makes it easy to be misdiagnosed. Herein, we report a rare case of small intestinal EMZL presentation as intussusception in a 32-year-old man. A colonoscopy performed at the local hospital revealed a pedicled polyp about 5 cm × 5 cm in size with a rough surface, and hyperemia was seen in the ileocecal region. He was admitted to our hospital for a polypectomy. A contrast-enhanced computed tomographic (CT) scan suggested ileocolic intussusception, which was subsequently confirmed by a colonoscopy in our hospital. Adult intussusception is relatively rare, with 90% of cases having a known causative mechanism and 40% of cases caused by primary or secondary malignancies. Therefore, we performed a laparoscopic-assisted right hemicolectomy for the patient. The resected specimen showed that the terminal ileum was intussuscepted into the ascending colon, and the intussusception was hyperemia and edema. A 2.5 cm × 2.5 cm × 1.5 cm mass was seen at the end of the intussusception. Postoperative pathology revealed that the mass was EMZL, partially transformed into a large B-cell lymphoma. The patient was transferred to the hematology department and completed a PET-CT showing postoperative manifestations of primary intestinal lymphoma, Lugano staging IE2. Although EMZL was an indolent lymphoma and the patient was in the early stages, the rituximab, cyclophosphamide, doxorubicin, vincristine, and prednisone (R-CHOP) regimen was given in view of the histological transformation. The patient is in regular follow-up. This was a rare case of small intestinal mass due to EMZL presented as intussusception in adults, which highlighted laparoscopic-assisted enterectomy as a potential therapeutic approach in the multidisciplinary collaborative therapy of small intestine EMZL.

## 1 Introduction

Extranodal marginal zone lymphoma of mucosa-associated lymphoid tissue (EMZL), also known as MALT lymphoma, is a low-grade lymphoma composed of small B cells of different morphology that invade multiple organs (1). It was first proposed in 1983 by P. Isaacson and D. H. Wright (2). EMZL most frequently occurs in the stomach, followed by ocular appendages, lungs, salivary glands, and other positions. The pathogenesis has not been elucidated and is linked to immune system dysregulation caused by prolonged immunological activation from chronic infections or autoimmune illnesses (3). Primary small intestinal EMZL is rare and lacks typical signs and symptoms, with great challenges in diagnosis and treatment. Intussusception is a disease in which part of the proximal bowel and its mesentery are embedded in the adjacent distal bowel. Intussusception is the main cause of acute abdomen in infants but is uncommon in adults. Unlike intussusception in children, 70%–90% of intussusception in adults has a clear etiology, of which about 40% is caused by primary or secondary malignancies, such as lymphoma, leiomyosarcoma, and adenocarcinoma (4, 5). To our knowledge, this is the first case of small intestinal EMZL with intussusception as the initial presentation, partially transforming into large B-cell lymphoma. Herein, we reported it to provide experience for clinical diagnosis and treatment. The presentation of this case adhered to the CARE checklists.

## 2 Case description

**Figure 1:**
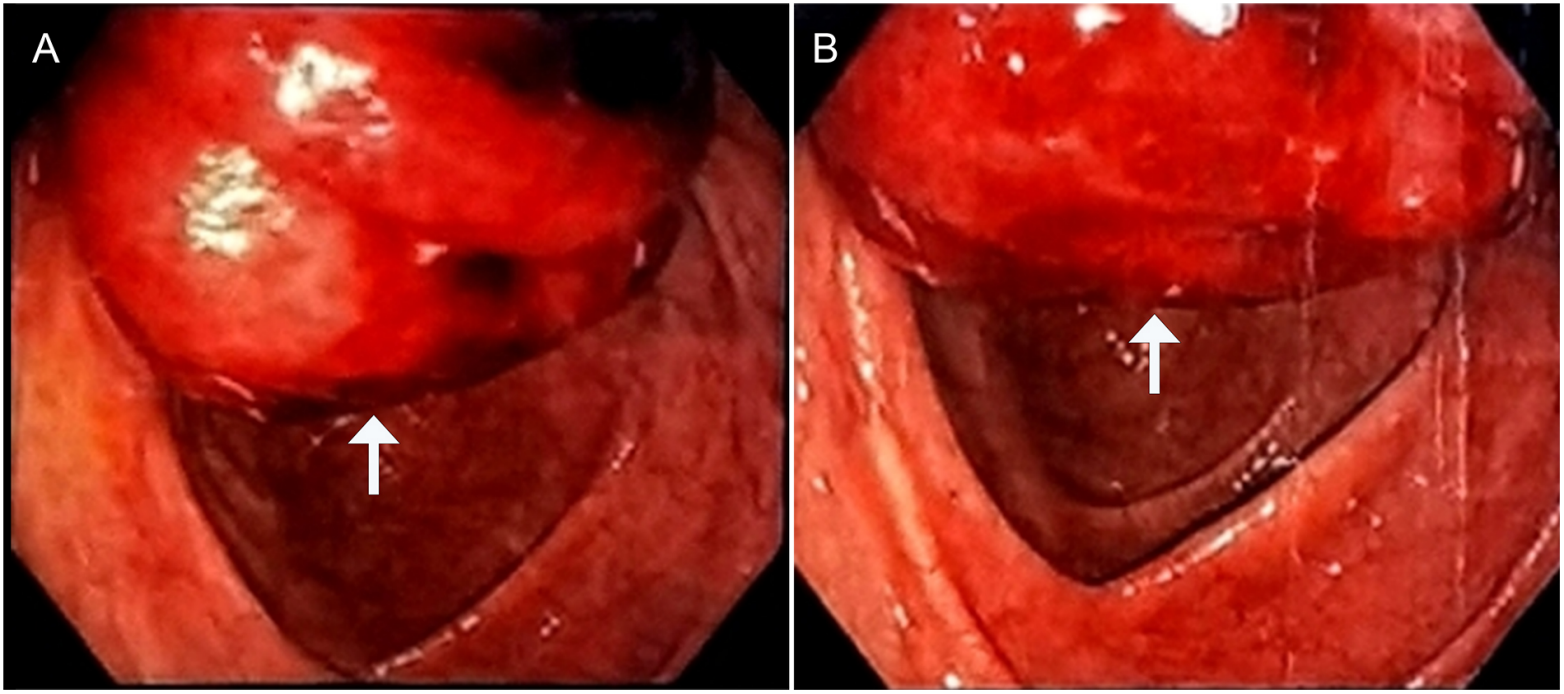
A colonoscopy performed at the local hospital reveals that a pedicled polyp with a rough surface and hyperemia was seen in the ileocecal region (the white arrows refer to the pedicled polyp).

**Figure 2:**
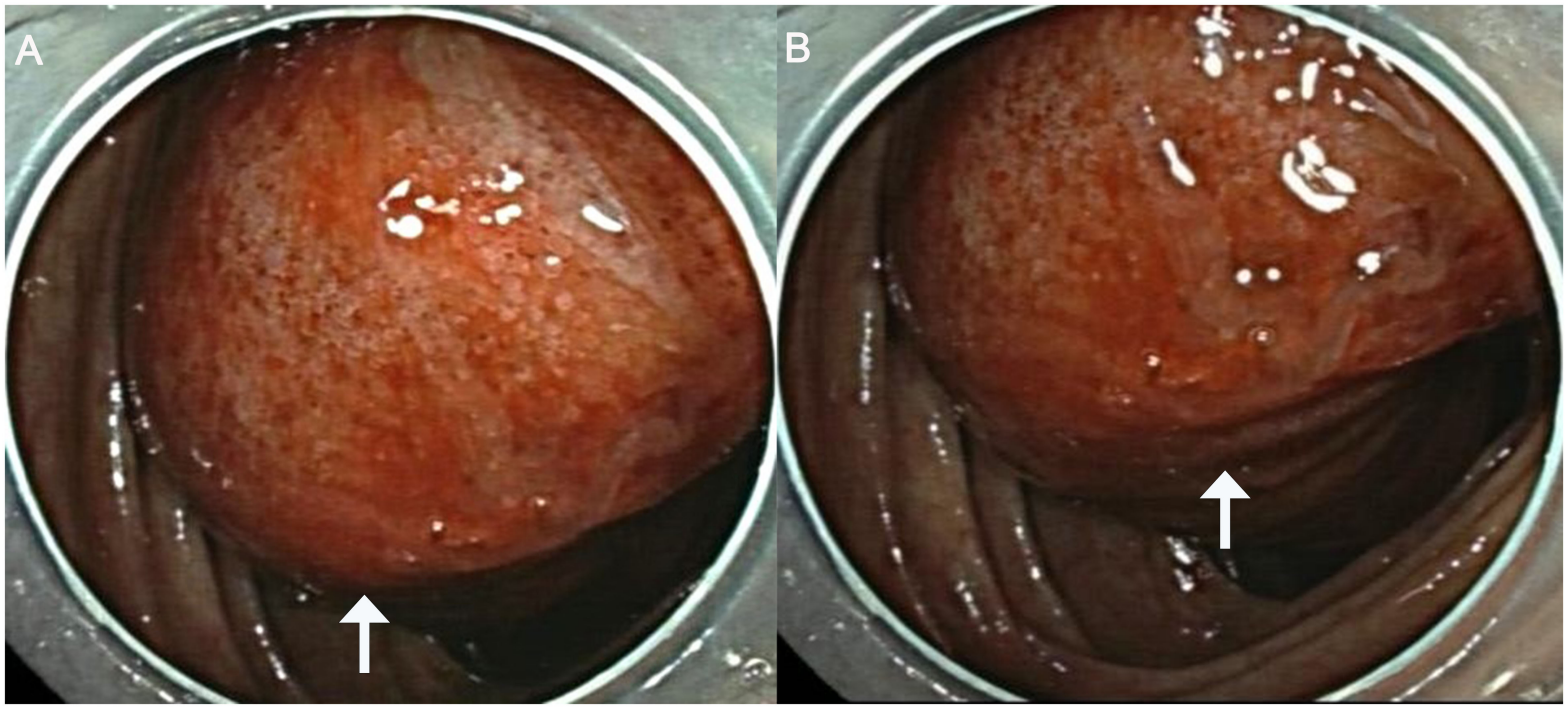
Results of the colonoscopy show a section of the terminal ileum with congestion and edema on the surface collapsing into the ascending colon (the white arrows refer to the terminal ileum collapsing into the ascending colon).

**Figure 3:**
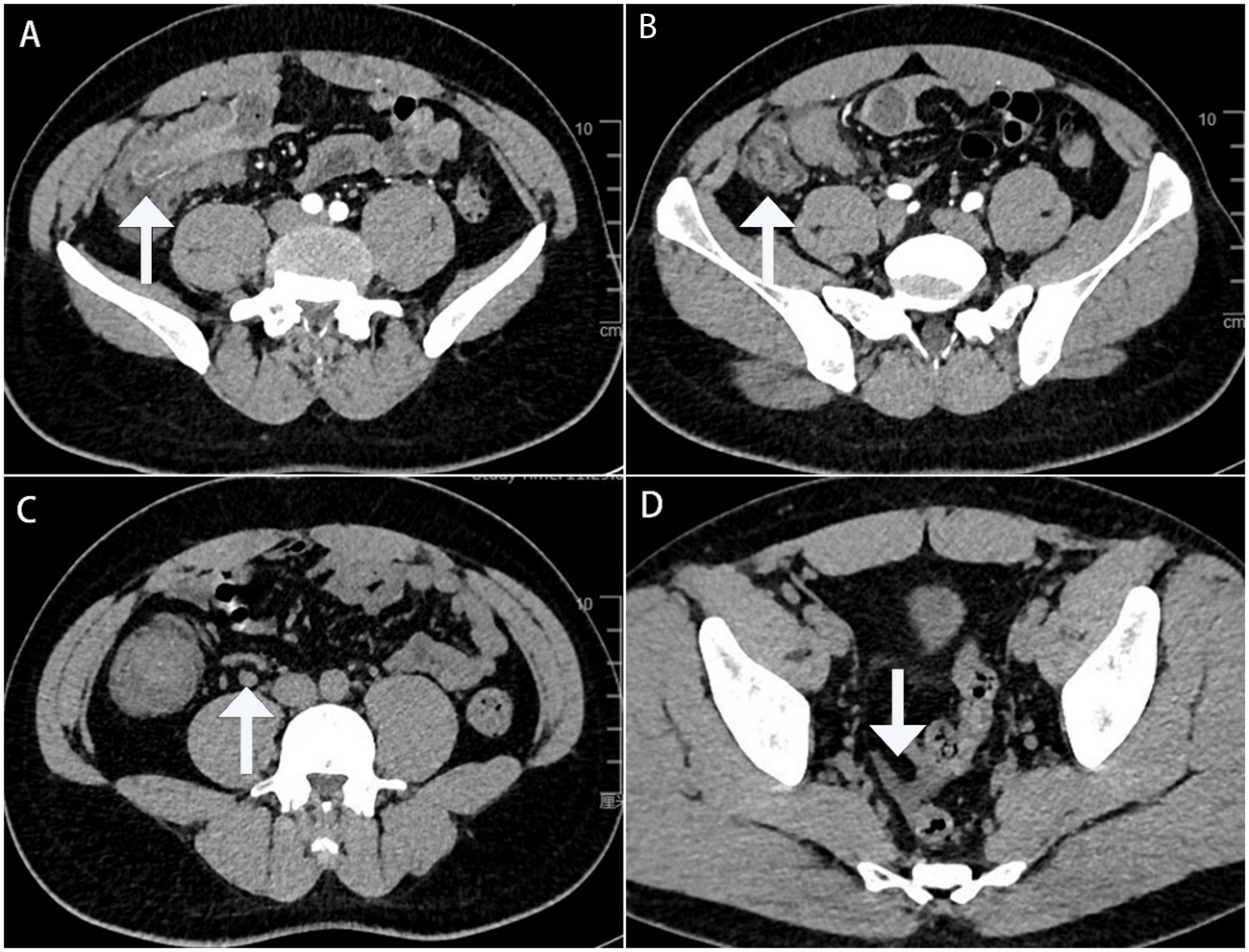
Enhanced computed tomography cross-section images show the terminal ileum collapsing into the ascending colon’s lumen: **(A)** with the typical “concentric circles” appearance (white arrows); **(B)** with the typical “sausage-like” sign appearance (white arrows); **(C)** with enlarged lymph nodes in the mesenteric region (white arrows); and **(D)** with a small amount of fluid in the pelvic cavity (white arrows).

**Figure 4:**
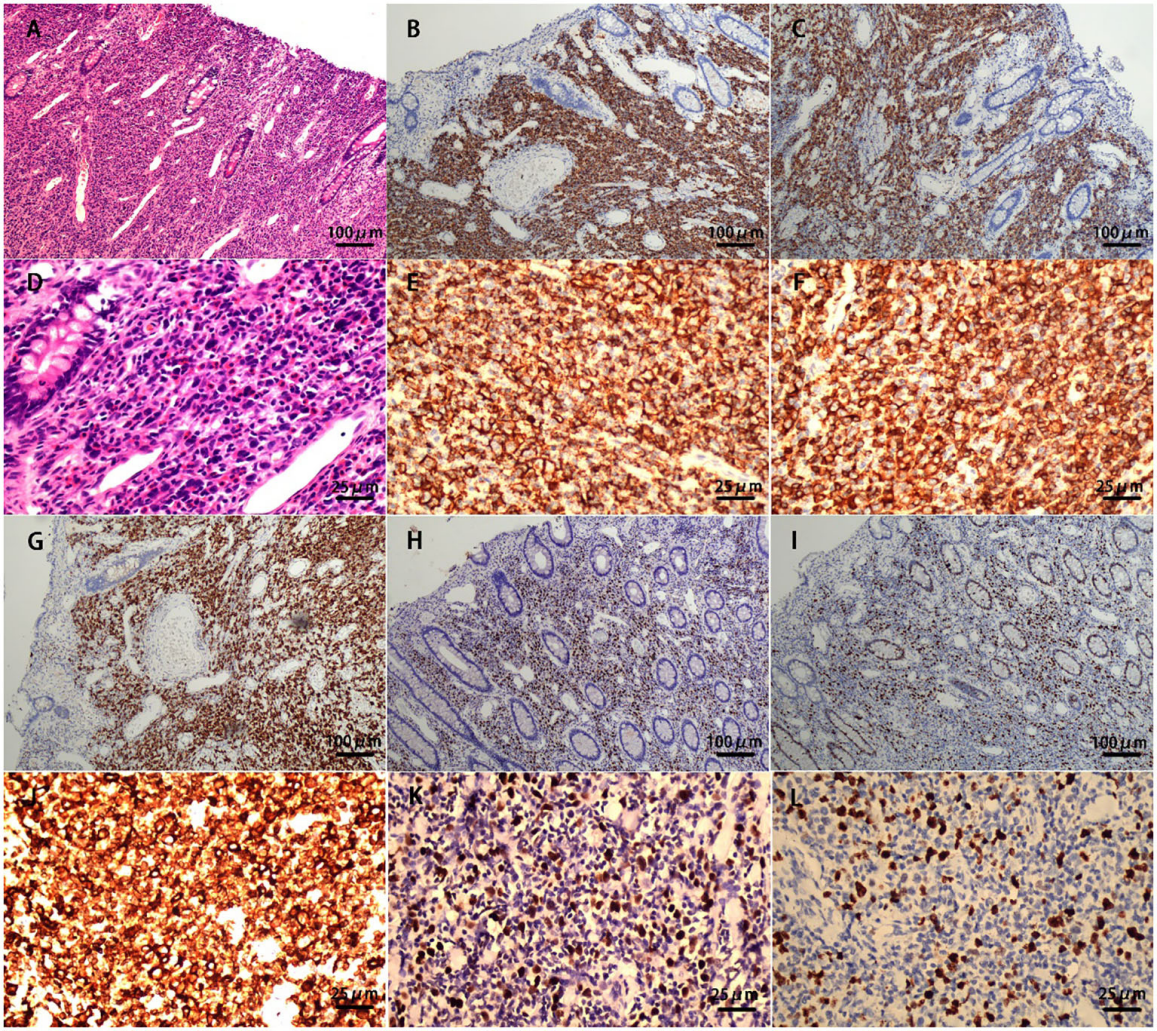
Postoperative pathology: **(A)** small bowel with a dense and diffuse lymphocytic infiltrate (HE, ×100); **(B)** atypical cells are diffusely positive for the marker CD20 (IHE CD20, ×100); **(C)** atypical cells are diffusely positive for the marker CD79a (IHE CD79a, ×100); **(D)** small bowel with a dense and diffuse lymphocytic infiltrate (HE, ×400); **(E)** atypical cells are diffusely positive for the marker CD20 (IHE CD20, ×400); **(F)** atypical cells are diffusely positive for the marker CD79a (IHE CD79a, ×400); **(G)** atypical cells are diffusely positive for the marker BCL-2 (IHE BCL-2, ×100); **(H)** atypical cells are diffusely positive for the marker BCL-6 (IHE BCL-6, ×100); **(I)** Ki-67 hot spots at about 40% (+) (IHE Ki-67, ×100); **(J)** atypical cells are diffusely positive for the marker BCL-2 (IHE BCL-2, ×400); **(K)** atypical cells are diffusely positive for the marker BCL-6 (IHE BCL-6, ×400); and **(L)** Ki-67 hot spots at about 40% (+) (IHE Ki-67, ×400).

A 32-year-old male patient was admitted to the local hospital for abdominal distension for more than 1 year and abdominal pain for 1 week. A colonoscopy (**Figure 1**) performed at the local hospital revealed a pedicled polyp about 5 cm × 5 cm in size with a rough surface and hyperemia was seen in the ileocecal region (biopsy). The pathology showed that the ileocecal region had chronic inflammation of the mucosal tissues with polypoid hyperplasia. A week later, the patient was admitted to our hospital for a polypectomy. The patient was previously in good health and had no family history of cancer. On clinical examination, pressing pain was located in the lower right abdomen without rebound pain or muscle tension; no palpable mass was in the abdomen; and no palpable liver or spleen was under the ribs. Blood testing was within normal limits. A chest computed tomographic (CT) examination showed no obvious abnormalities. A colonoscopy (**Figure 2**) was performed in our hospital for polypectomy, but it revealed a section of the small intestine with congestion and edema on the surface was embedded in the ascending colon. The ileocecal flap was not discernible. The abdominal contrast-enhanced CT scan (**Figure 3**) showed that the wall of the ileocecal region was blurred, and the mesentery and end of the ileum were collapsed into the ascending colon’s lumen, presenting “sausage-like” signs and “concentric circles” along with a few oozing shadows and small lymph nodes surrounding it. Different-sized lymph nodes were observed in the mesenteric region. A small amount of fluid was in the pelvic cavity. Considering that 90% of adults with intussusception involve a pathologic lead point, about 40% of which are malignancies (6), laparoscopic-assisted right hemicolectomy with lymph node dissection was performed. Intraoperative findings were as follows: no lesions in the pelvic and abdominal cavity; terminal ileum imbedded in the ascending colon; mild adhesions in the right lower abdomen; and a small amount of yellowish-clear ascites. The resected specimen showed that the terminal ileum intussuscepted into the ascending colon; intussusception was hyperemia and edema; and a 2.5 cm × 2.5 cm × 1.5 cm mass was seen at the end of the intussusception. Postoperative pathology (**Figure 4**) showed the right hemicolon resection specimen: B-cell lymphoma of the terminal ileum. Combined with immunohistochemical markers and T and B lineage gene rearrangement tests, it was considered to be lymphoma of small B-cell origin (extranodal marginal zone B-cell lymphoma), partially transformed into large B-cell lymphoma (moderately aggressive). The tumor tissue infiltrated the whole layer of the intestinal wall; there was no tumor tissue involvement in the lateral margins of the intestinal canal at the two ends and the omental tissues; and there was no metastasis of the tumor tissue to periampullary lymph nodes (0 out 17 lymph nodes removed during surgery). The remaining intestinal mucosa showed mild chronic inflammation with localized intussusception. Tumor cells were immunohistochemically shown to be positive for CD20, CD79a, BCL 2, and BCL 6; focal positive for CD23 and CD10; and negative for CD3, CD5, CD43, CD4, CD8, cyclin D1, SOX11, MUM 1, TdT, CD30, or CD15. Ki-67 showed hot spots at about 40% (+). The rest of the findings were as follows: *in situ* hybridization: EBER (-). PCR: B lineage gene rearrangement detection: B and D tubes monoclonal bands detected (+); IGK: monoclonal band detected (+); PCR: T lineage gene rearrangement detection: TCRB: monoclonal band detected (-); TCRD: monoclonal band detected (-); and TCRG: monoclonal band detected (-).

After the operation, he was referred to the hematology department for adjuvant chemotherapy. Whole-body PET-CT was consistent with the postoperative manifestations of the primary intestinal lymphoma, Lugano staging IE2; systemic bone marrow FDG metabolism is mildly increased, suggesting a reactive hyperplasia; mesenteric lymph nodes in the operation area; and FDG metabolism is not abnormally increased. Bone marrow aspiration with lymphocyte immunophenotyping showed no abnormal findings. The results of the genetic examination showed that no mutation in the B-cell lymphoma-related gene locus was found. Although the patient is in the early stages of EMZL, the patient underwent a rituximab, cyclophosphamide, doxorubicin, vincristine, and prednisone (R-CHOP) regimen at a frequency of every 21 days in view of the histological transformation into large B-cell lymphoma. The fourth cycle of treatment has just been completed and was well-tolerated, with the patient experiencing only mild hair loss and numbness in the fingers. At present, the blood routine and liver and kidney functions are basically normal. The patient is in regular follow-up.

## 3 Discussion

MZL is a group of B-cell malignant neoplastic diseases originating in the follicular marginal zone that can occur in lymph nodes and extra-lymph nodes, accounting for 5%–15% of non-Hodgkin lymphoma (NHL) in western countries (7). It is the second most common subtype of B-cell NHL in Asia [after diffuse large B-cell lymphoma (DLBCL)] and the fourth most common subtype in the USA (after DLBCL, follicular, and Burkitt lymphoma) (8). The 5th edition of the WHO Classification classifies MZL into four types: mucosa-associated lymphoid tissue extranodal marginal zone lymphoma (EMZL), lymph node MZL, primary cutaneous marginal zone lymphoma, and pediatric marginal zone lymphoma in 2022, which is different from the 4th edition of the WHO Classification of Haematolymphoid Tumours: Lymphoid Neoplasms (9). EMZL is an uncommon cause of primary gastrointestinal lymphoma and can involve multiple organs, such as the stomach, ocular appendages, lungs, and salivary glands. Primary small intestinal EMZL is rare and is slightly more common in men (men:women ratio of 1.5:1), and the incidence increased exponentially with age, with a median age at diagnosis of 59 years old (8).

EMZL is associated with chronic antigenic stimulation caused by infectious agents or autoimmune processes and can occur at widely different sites, with gastric EMZL accounting for >30% of cases and other common sites including ocular appendages, salivary glands, and lungs, with varying site-specific etiologies (10). Chronic *Helicobacter pylori* gastritis was reported as a susceptibility factor for gastric EMZL; *Campylobacter jejuni* for small intestinal EMZL; *Achromobacter xylosoxidans* possibly for lung; and *Borrelia burgdorferi* for cutaneous MZLs (11–13). Some studies have also shown that hepatitis C virus (HCV) infection is associated with a portion of B-cell NHL, including EMZL (14, 15). Autoimmune diseases such as Sjogren syndrome and Hashimoto’s thyroiditis are associated with an increased risk of salivary gland and thyroid EMZL, respectively. In addition, a family history of EMZL suggests its genetic susceptibility (3).

The clinical presentation of EMZL varies widely, depending on the site of involvement. The symptoms of small intestinal EMZL vary from abdominal pain, chronic bleeding, weight loss, and intestinal obstruction to no symptoms only detected by abdominal CT or endoscopy (16, 17). The patient in this case report presented with chronic abdominal distension and abdominal pain, and then he completed an abdominal CT and colonoscopy considering ileocolic intussusception. Considering that the patient did not present with acute intestinal obstruction, we chose a minimally invasive approach. We know that 90% of adult intussusceptions had underlying etiologies, and 40% of them were malignancies. The principles of oncology surgery must be followed, and appropriate resection of the bowel and lymph node dissection should be performed depending on the location (18), so we chose laparoscopic-assisted right hemicolectomy for this patient because the lesion was in the ileocecum. Postoperative pathology revealed B-cell lymphoma of the terminal ileum. Combined with CD20 (3+), CD79a (3+), and BCL-2 (3+), it is considered EMZL. However, BCL-6 is partially expressed and the hotspot area of Ki-67 is approximately 40%, considering it partially transformed into large B-cell lymphoma. Several studies have shown that patients with aggressive lymphoma have shorter progression-free survival and overall survival (19, 20). Although small intestinal EMZL with intussusception has been previously reported (**Table 1**) (21–24), histological transformation in this case is very rare. At present, the mechanism related to transformation remains unclear. Continuous exposure to foreign antigens lowers the physiological threshold for triggering proliferation, which may predispose them to histological transformation. Some studies have shown that the transformation into higher-grade lymphoma was associated with high lactate dehydrogenase and multiple lymph node involvement at diagnosis, failure to achieve a complete response after initial treatment (25), multiple mucosal site involvement, CD5 expression, and complex karyotypes (26). However, this patient had none of the risk factors.

**Table 1:** A summary of four cases of intussusception due to EMZL reported in the literature from 1995 to 2024.

Reference	Year	Age	Sex	Complaint	Palpable mass	Diagnostic tools	Location	Surgical procedure	Medical approach	Follow-up
(19)	1995	44	F	AP + anemia	Positive	Col	Terminal ileum	Right hemicolectomy	NS	NS
(20)	2013	35	F	AP	Positive	US	Terminal ileum	Right hemicolectomy	ChT	NS
(21)	2016	83	F	AP + diarrhea	Positive	CT	Terminal ileum	Seg Resection	NS	NS
(22)	2022	78	M	AP + weight loss	Negative	MRI	Ileum	Seg Resection	ChT (R-CHOP)	4 months

AP, abdominal pain; col, colonoscopy; NS, not stated; US, ultrasonography; ChT, chemotherapy; CT, computed tomography; Seg: segmental; and MRI, magnetic resonance imaging.

If EMZL is suspected, clinicians should obtain the largest biopsy specimen possible, as small specimens may not provide enough tissue. The diagnosis of small intestine EMZL remains difficult because most tumors are not detectable by gastroscopy and colonoscopy. Only balloon enteroscopy can be used to detect lesions and determine the histological type by biopsy. The literature regarding the endoscopic features of small intestinal EMZL is not sufficient. In a meta-analysis of 415 cases of small intestinal lymphoma in the Chinese population, 97 cases of EMZL were identified, of which 48% were described as ulcers and 17% as masses (27). Another review included 53 cases of small intestinal EMZL-reported endoscopy in 23 patients. Endoscopic manifestations of ulcerative and erosive lesions were found in 82.6% of patients (8). Some cases of gastrointestinal tract obstruction or intussusception require emergent surgical intervention, and postoperative pathology can also identify the tumor type (23, 28). Consistent with other published cases (**Table 1**). The case reported in this study underwent emergency surgery due to intussusception, and postoperative gross specimens showed that the starting point of intussusception was a mass.

Most cases present with local lesions (I–II) at diagnosis of EMZL, and about 25% of cases present with multifocal single-organ involvement or diffuse extranodal involvement (IV), especially in extragastric EMZL (29). Both gastrointestinal and non-gastrointestinal EMZL have good prognoses, with 5-year overall survival (OS) rates above 90% and 10-year OS rates of 75% to 80% (16). An observational study including 321 MZL patients showed that disease progression within 24 months after initial treatment with MZL was a predictor of reduced OS, compared to patients whose disease did not progress within 24 months (3-year OS, 95%), and patients whose disease progressed within 24 months had a 3-year OS of only 53%, with a hazard ratio of 19.5 (95% confidence interval, 8.4–45) (30). Due to the risk of occult spread, extensive initial staging assessment is required regardless of the site of onset, especially before antibiotic therapy or radiotherapy (31). The EMZL staging of the gastrointestinal tract depends mainly on the 2014 Lugano classification based on CT, MRI, or PET-CT (32). The lymphoma in this case was confined to the small intestine, which infiltrated into the serous membrane without lymph node metastasis or distant metastasis, so for this patient, the stage was IE2 according to the Lugano classification.

The treatment and monitoring of EMZL require multidisciplinary collaboration, including gastroenterologists, hematologists, oncologists, and doctors in other related specialties. All gastric EMZL patients should receive <i>H. pylori</i> eradication therapy, regardless of stage, and most patients can achieve complete remission. Studies showed that with the increase in <i>H. pylori</i> eradication rate, the proportion of <i>H. pylori</i>-negative gastric EMZL began to increase, which accounted for 40% of all gastric EMZL patients (33, 34). The value of anti-infective therapy in other EMZLs is poorly understood. For patients whose lymphoma has not subsided after antibiotic therapy, radiation therapy (RT) and systemic tumor therapy should be used, depending on the stage of the disease. In <i>H. pylori</i>-negative patients, if no signs of lymphoma regression are seen in a repeat endoscopic evaluation 3–6 months after antibiotic treatment, RT should be considered (35). Chemotherapy, immunotherapy, or chemotherapy combined immunotherapy may be used for patients with EMZL with contraindications to radiotherapy, failure of antibiotics, surgery or radiotherapy, histological transformation, and symptomatic systemic disease. Patients whose disease has undergone histological transformation into large B-cell lymphoma should be treated with chemo-immunotherapy as DLBCL (7). Although the disease of the patient in this case report was staged in Lugano IE2, the pathologic findings suggested EMZL of the ileum and partial transformation into DLBCL, which suggested a poor prognosis and a shorter OS. Therefore, this case was treated with R-CHOP chemo-immunotherapy after surgery, the first-line treatment for DLBCL (36). He had completed four cycles of R-CHOP so far, with clinical remission.

## 4 Conclusion

MZLs are indolent lymphomas with great heterogeneity in etiology, clinical manifestations, diagnosis, and treatment. EMZL is the most common subtype of MZL. The symptoms of small intestinal EMZL include abdominal pain, chronic bleeding, loss of weight, and intestinal obstruction. A few patients present with intussusception. For adult intussusception, although preoperative imaging data do not indicate a tumor, performing resection and lymph node dissection are still needed according to the principle of tumorectomy. After an operation, the next treatment plan is determined according to the pathologic type.

## Data availability statement

The original contributions presented in the study are included in the article/supplementary material. Further inquiries can be directed to the corresponding authors.

## Ethics statement

This article was a case report and the patient has signed informed consent for publication. The studies were conducted in accordance with the local legislation and institutional requirements. The participants provided their written informed consent to participate in this study. Written informed consent was obtained from the individual(s) for the publication of any potentially identifiable images or data included in this article.

## Author contributions

BD: Conceptualization, Writing – original draft. JZ: Writing – review & editing. FZ: Writing – review & editing. ZW: Supervision, Writing – review & editing. YD: Supervision, Writing – review & editing.
